# *Spirulina maxima* Extract Reduces Obesity through Suppression of Adipogenesis and Activation of Browning in 3T3-L1 Cells and High-Fat Diet-Induced Obese Mice

**DOI:** 10.3390/nu10060712

**Published:** 2018-06-01

**Authors:** Young-Jin Seo, Kui-Jin Kim, Jia Choi, Eun-Jeong Koh, Boo-Yong Lee

**Affiliations:** Department of Food Science and Biotechnology, College of Life Science, CHA University, Seongnam, Kyeonggi 463-400, Korea; youngjinseo92@gmail.com (Y.-J.S.); kuijin.kim@gmail.com (K.-J.K.); wldk3176@gmail.com (J.C.); kej763@naver.com (E.-J.K.)

**Keywords:** obesity, *Spirulina maxima*, adipogenesis, browning, 3T3-L1 cells, HFD-induced obese mice

## Abstract

Obesity predisposes animals towards the metabolic syndrome and diseases such as type 2 diabetes, atherosclerosis, and cardiovascular disease. *Spirulina maxima* is a microalga with anti-oxidant, anti-cancer, and neuroprotective activities, but the anti-obesity effect of *Spirulina maxima* 70% ethanol extract (SM70EE) has not yet been fully established. We investigated the effect of SM70EE on adipogenesis, lipogenesis, and browning using in vitro and in vivo obesity models. SM70EE treatment reduced lipid droplet accumulation by the oil red O staining method and downregulated the adipogenic proteins C/EBPα, PPARγ, and aP2, and the lipogenic proteins SREBP1, ACC, FAS, LPAATβ, Lipin1, and DGAT1 by western blot analysis. In addition, the index components of SM70EE, chlorophyll a, and C-phycocyanin, reduced adipogenesis and lipogenesis protein levels in 3T3-L1 and C3H10T1/2 cells. High-fat diet (HFD)-fed mice administered with SM70EE demonstrated smaller adipose depots and lower blood lipid concentrations than control HFD-fed mice. The lower body mass gain in treated SM70EE-administrated mice was associated with lower protein expression of adipogenesis factors and higher expression of AMPKα-induced adipose browning proteins PRDM16, PGC1α, and UCP1. SM70EE administration ameliorates obesity, likely by reducing adipogenesis and activating the thermogenic program, in 3T3-L1 cells and HFD-induced obese mice.

## 1. Introduction

Obesity is one of the leading preventable causes of death and is becoming an increasingly serious social problem worldwide. Obesity can lead to metabolic disease, including hypertension, type 2 diabetes, hyperlipidemia, and arteriosclerosis [1], and is caused by an imbalance between energy intake and expenditure in adipose tissue [2]. There are two distinct types of adipose tissue in mammals: white adipose tissue (WAT) and brown adipose tissue (BAT). WAT stores excess energy in the form of triglyceride (TG), which can be lipolyzed to liberate glycerol and free fatty acids [3]. BAT specializes in energy expenditure through induction of uncoupling protein 1 (UCP1) expression [4,5]. Recently, it was established that UCP1 can be expressed in WAT adipocytes that are referred to as ‘beige’ or ‘brite (brown-in-white)’, representing ‘inducible BAT’ [6]. These adipocytes undergo a browning process, which converts preadipocytes into beige adipocytes [7], which also play a crucial role in the regulation of energy.

Adipogenesis is the cellular differentiation process that transforms preadipocytes into mature adipocytes, involving dramatic changes in adipocyte morphology, and which can be artificially induced using 3-isobutyl-1-methylxanthine (IBMX), dexamethasone, and insulin. Many transcription factors regulate this process, including CCAAT/enhancer-binding protein alpha (C/EBPα), peroxisome proliferator-activated receptor gamma (PPARγ), and adipocyte protein 2 (aP2) [8]. In addition, lipogenic related enzymes, such as fatty acid synthase (FAS) and diacylglycerol acyltransferases (DGAT), play important roles in lipid accumulation during adipogenesis [9]. High expression levels of lipogenic genes, such as acetyl-CoA carboxylase (ACC), FAS, lysophosphatidic acid acyltransferase (LPAAT), lipin, and DGAT enhance adipogenesis and obesity [10,11,12], whereas LPAATβ and lipin1-deficient cells do not express adipogenic marker proteins, such as C/EBPα and PPARγ, during adipocyte differentiation [13,14].

AMP-activated protein kinase alpha (AMPKα) activation has been suggested to inhibit adiposity, reducing expression of C/EBPα and PPARγ in 3T3-L1 cells and high-fat diet (HFD)-induced obese mice [15,16]. AMPKα is induced by an increase in the cellular AMP/ATP ratio, which regulates energy production or consumption. Recently, studies have revealed that AMPKα can directly interact with PPARγ coactivator 1 alpha (PGC1α) [17], which has been shown to be necessary for mitochondrial and thermogenic activity [18]. Thermogenesis is induced by brown adipocyte-specific markers, including PR domain containing 16 (PRDM16), PGC1α, and UCP1 [7].

It has also been reported that a variety of phytochemicals can affect body weight, adipocyte differentiation, and the conversion of white fat cells to brown or beige adipocytes [19]. *Spirulina maxima* is a microalga that is rich in essential nutrients and contains pigment proteins such as chlorophyll a and C-phycocyanin [20,21,22,23]. Several reports have proposed that chlorophyll a and C-phycocyanin are index components of *Spirulina maxima* [24,25]. Recently, many studies have demonstrated that *Spirulina maxima* has anti-oxidant, anti-cancer, and neuroprotective properties [20,26,27]. An index compound of *Spirulina maxima*, chlorophyll a, has anti-oxidant and anti-inflammatory activities [28], while a second, C-phycocyanin, has anti-oxidant, anti-inflammatory, and anti-diabetic potential [29,30]. However, it is not known whether *Spirulina maxima* has anti-obesity effects. The aim of this study was to investigate the anti-obesity and browning effects of *Spirulina maxima* 70% ethanol extract (SM70EE) in vitro and in vivo. In addition, we investigated whether chlorophyll a and C-phycocyanin have effects as index components on adipogenesis in vitro. We show that *Spirulina maxima* extracts can ameliorate obesity-related markers by modulating adipogenesis and browning of adipocyte.

## 2. Materials and Methods

### 2.1. Experimental Materials

SM70EE was acquired from the Korea Institute of Ocean Science & Technology, where it was produced by a two-phase method of extraction. In the first step, *Spirulina maxima* was ultrasound-extracted in 70% ethanol at room temperature for ~8 h. The product was then extracted at 65–70 °C for ~4 h and the extract was analyzed [24]. The composition of SM70EE are shown in Table 1. Chlorophyll a was purchased from Toronto Research Chemicals (Toronto, ON, Canada) and C-phycocyanin was acquired from Xi’an Day Natural Inc. (Pudong, Shanghai, China). The chlorophyll a and C-phycocyanin content of the SM70EE was quantified by high-performance liquid chromatography photo-diode array (HPLC-PDA) analysis, as previously described [24]. The chlorophyll a and C-phycocyanin are contained in SM70EE at approximately 5% and 10%, respectively. Dulbecco’s modified Eagle’s medium (DMEM), bovine calf serum (BCS), fetal bovine serum (FBS), penicillin-streptomycin (P/S), insulin, and trypsin-EDTA were purchased from Gibco (Gaithersburg, MD, USA). Dexamethasone, isopropanol, oil red O, and chemical reagents were mainly purchased from Sigma-Aldrich (St Louis, MO, USA). Primary antibodies specific for C/EBPα, PPARγ, aP2, SREBP1, ACC, FAS, LPAATβ, Lipin1, DGAT1, PGC1α, α-tubulin, GAPDH (Santa Cruz Biotechnology, Dallas, TX, USA), p-AMPKα (Cell signaling, Beverly, MA, USA), PRDM16, and UCP1 (Abcam, Cambridge, UK) were purchased.

### 2.2. Cell Culture

Mouse 3T3-L1 preadipocytes and C3H10T1/2 cells were obtained from the ATCC (Manassas, VA, USA) and were maintained in DMEM containing 10% BCS and 1% penicillin/streptomycin (P/S) at 37 °C in a 5% CO_2_ incubator. To induce adipogenesis, C3H10T1/2 cells were incubated in DMEM and BMP4 (10 μg/mL) for ~3 days, until they became confluent. After this, the preadipocytes were cultured in DMEM containing 10% FBS, 0.5 mM IBMX, 1 μM dexamethasone, and 4 μg/mL insulin for 2 days. To stimulate browning of adipocytes, the differentiation medium was supplemented with 50 nM triiodothyronine and 1 μM rosiglitazone for 2 days. The adipocyte culture medium consisted of DMEM containing 10% FBS and 4 μg/mL insulin, and was changed every 2 days.

### 2.3. Cell Viability Assay

3T3-L1 preadipocytes (10^4^ cells/well) were incubated in DMEM containing 10% BCS overnight in 96-well plates. The cells were then treated with SM70EE (0, 6.25, 12.5, 25, 50, 100, or 200 μg/mL), chlorophyll a, or C-phycocyanin (0, 0.625, 1.25, 2.5, 5, 10, or 20 μg/mL) for 24 h. Twenty microliters of MTT solution was added to each well and the cells were incubated at 37 °C for a further 3 h. Then, the MTT-containing media was gently aspirated, and 100 μL DMSO was added to extract intracellular formazans, which were quantified by ELISA, using a Wallac 140 Victor spectrophotometer (Perkin Elmer, Boston, MA, USA) at 570 nm.

### 2.4. Oil Red O Staining

Differentiated 3T3-L1 adipocytes were fixed with 10% formalin in PBS for 1 h and washed twice with 60% isopropanol. The fixed cells were then stained using oil red O solution for 30 min and washed with distilled water. After drying, the fixed cells were imaged by scanner. The oil red O solution taken up by the cells was then extracted using 100% isopropanol and its optical intensity was measured at 490 nm.

### 2.5. Animal Studies

Male ICR mice were purchased from Orient Bio Co. (Gapyeong, Kyeonggi, Korea) at 4 weeks of age. The animal study was approved by the Institutional Animal Care and Use Committee (IACUC) of CHA University (Approval Number 170091). Mice were kept under temperature and humidity-controlled conditions at a 12 h light/dark cycle. After a 1-week acclimation period, they were fed a chow diet (Envigo, Huntingdon, Cambridgeshire, UK), a 60 kcal% fat HFD (Central Lab Animal Inc., Seoul, Korea), or a HFD supplemented with SM70EE for 6 weeks (Table 2). The vehicle of the CHOW and HFD group administered SM70EE 0 mg/kg/day. During the experiment, body mass, fasting blood glucose, and food intake were measured weekly. After this period, the mice were starved overnight and euthanized, and blood and tissue samples were collected.

### 2.6. Blood Parameter Analysis

Mice were sacrificed by CO_2_ asphyxiation. Blood was collected by cardiac puncture and placed into blood collecting tubes aseptically. To collect serum, blood was coagulated for 1 h at room temperature and then centrifuged at 13,000× *g* for 15 min at 4 °C. Serum levels of TG, total cholesterol, low-density lipoprotein (LDL)-cholesterol, and high-density lipoprotein (HDL)-cholesterol were measured using kits supplied by Roche (Mannheim, Germany) using the manufacturers’ protocols.

### 2.7. Preparation of the Stromal Vascular Fraction

Stromal vascular fractions (SVFs) were isolated by digestion of subcutaneous adipose tissue from C57BL/6 mice for 1 h in PBS containing 10 mM CaCl_2_, 1.5 units/mL collagenase D, and 2.4 units/mL dispase. The process was stopped by the addition of DMEM/F12 containing 10% FBS, and the digest was then filtered through a 70-μm cell strainer. The cell suspension was then seeded into 10 cm plates, and after 2 h the medium was changed to remove red blood cells and other residues. Differentiation of the isolated SVF cells was then induced using an induction medium, as described previously [31].

### 2.8. Western Blotting

Cells and tissues were lysed in lysis buffer (iNtRON Biotechnology Inc., Seoul, Korea) containing protease and phosphatase inhibitors (Sigma-Aldrich). Protein concentration was determined using a Bradford assay kit (Bio-Rad Laboratories, Hercules, CA, USA). Equal amounts of protein were separated by sodium dodecyl sulfate-8–12% polyacrylamide gel electrophoresis and transferred to polyvinylidene fluoride membranes. After blocking with 5% skim milk for 1 h at room temperature, the membranes were incubated with primary antibodies overnight at 4 °C, followed by incubation with horseradish peroxidase-conjugated secondary antibodies (1: 5000) for 2 h at room temperature. Finally, protein bands were visualized with an enhanced chemiluminescence solution and analyzed using a Chemi-doc (Bio-Rad Laboratories).

### 2.9. Statistical Analysis

Data are presented as mean ± standard deviation (SD). One-way ANOVA with Duncan’s test was used to assess differences between groups (SPSS, Chicago, IL, USA). *p* < 0.05 was regarded as representing statistical significance, and for multiple comparisons, statistical differences among the groups are indicated below using ‘a, b, c, and d’.

## 3. Results

### 3.1. SM70EE, Chlorophyll a, and C-Phycocyanin Inhibit Lipid Accumulation in 3T3-L1 Cells

**Figure 1:**
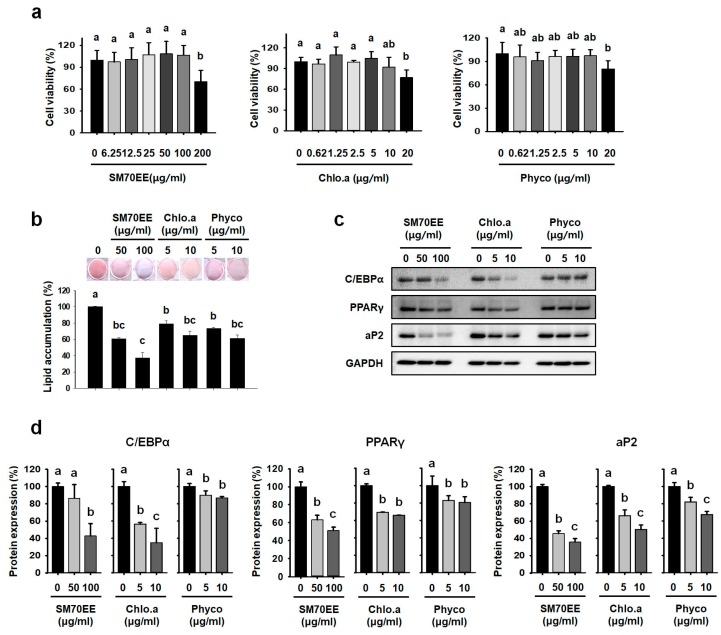
SM70EE, chlorophyll a, and C-phycocyanin inhibit adipogenic differentiation in 3T3-L1 cell. (**a**) Effect of SM70EE, chlorophyll a, and C-phycocyanin on cell viability in 3T3-L1 preadipocytes, determined using a 24-h MTT assay. (**b**) Effect of SM70EE, chlorophyll a, and C-phycocyanin on lipid accumulation, determined using oil red O staining, in 3T3-L1 adipocytes. A differentiation-inducing cocktail with or without SM70EE, chlorophyll a, or C-phycocyanin was added to 3T3-L1 adipocytes for 8 days. (**c**) Western blot analysis of adipogenic markers (C/EBPα, PPARγ, and aP2) after 8 days of incubation of 3T3-L1 adipocytes in differentiation medium. (**d**) The specific bands in (**c**) were quantified and are presented as graphs. Data are expressed as mean ± SD (*n* = 3). Values with different letters are significantly different, *p* < 0.05 (a > b > c).

To evaluate the cytotoxicity of SM70EE and index components chlorophyll a and C-phycocyanin, we performed an MTT assay in 3T3-L1 preadipocytes. As shown in Figure 1a, SM70EE at 200 μg/mL impaired cell viability; therefore, 50 and 100 μg/mL SM70EE were used in further investigations. We also showed that chlorophyll a and C-phycocyanin have no significant cytotoxicity when administered at up to 10 µg/mL to mice.

To evaluate the effects of SM70EE, chlorophyll a, and C-phycocyanin, 3T3-L1 cells were cultured in adipocyte differentiation media for 8 days in the presence or absence of SM70EE, chlorophyll a, or C-phycocyanin. We then undertook oil red O staining and western blot analysis to assess their effects on adipogenic differentiation. As shown in Figure 1b, SM70EE had a dose-dependent effect to reduce oil red O staining versus that of control 3T3-L1 cells. Chlorophyll a and C-phycocyanin reduced lipid droplet accumulation in 3T3-L1 adipocytes compared to control differentiated adipocytes. As shown in Figure 1c,d, SM70EE, chlorophyll a, and C-phycocyanin blocked expression of the differentiation-induced proteins including C/EBPα, PPARγ, and aP2 during 3T3-L1 differentiation. However, SM70EE suppressed the expression of late stage adipogenic regulators more profoundly than chlorophyll a and C-phycocyanin in these cells.

### 3.2. SM70EE, Chlorophyll a, and C-Phycocyanin Regulate Lipogenesis Pathway Enzymes in 3T3-L1 cells

**Figure 2:**
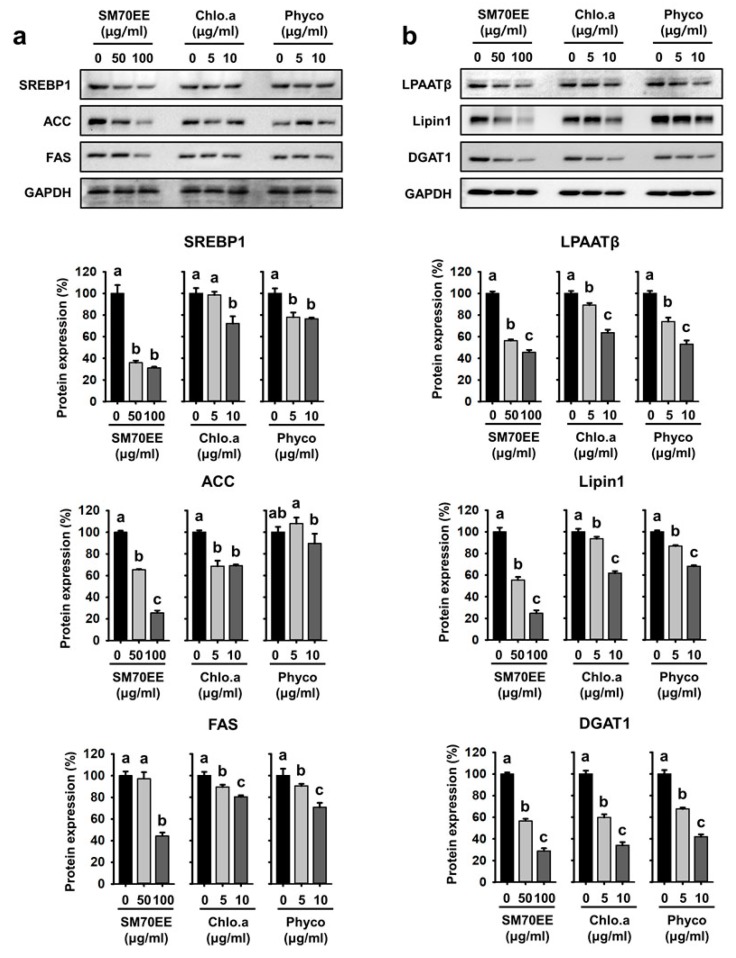
SM70EE, chlorophyll a, and C-phycocyanin regulate lipogenesis pathway enzyme expression in 3T3-L1 cells. (**a**) Western blots of SREBP1, ACC, and FAS protein expression after differentiation for 8 days. Specific bands were quantified and these are presented as graphs. (**b**) Representative western blots of lipogenic proteins (LPAATβ, lipin1, and DGAT1) in 3T3-L1 adipocytes. Data are expressed as mean ± SD (*n* = 3). Values with different letters are significantly different, *p* < 0.05 (a > b > c).

To elucidate the mechanisms of the lipogenic effect of SM70EE, chlorophyll a, and C-phycocyanin, we analyzed the expression levels of the lipogenic genes SREBP-1c, ACC, FAS, LPAATβ, lipin1, and DGAT1 by western blotting. Culture in differentiation media enhanced the expression of SREBP-1c, its downstream target, PPARγ, and the lipogenic enzymes ACC and FAS in adipocytes [32,33]. As shown in Figure 2a, protein levels of SREBP1, ACC, and FAS were lower in the presence of SM70EE. Consistent with the above data, addition of the SM70EE index compounds chlorophyll a or C-phycocyanin dramatically reduced the expression of the lipogenic markers LPAATβ, lipin1, and DGAT1 (Figure 2b). Taken together, our data show that SM70EE, chlorophyll a, and C-phycocyanin significantly suppress lipogenesis by reducing lipogenic gene expression during the differentiation of 3T3-L1 cells.

### 3.3. SM70EE, Chlorophyll a, and C-Phycocyanin Reduce the Expression of Markers of Adipogenesis and Lipogenesis in C3H10T1/2 Cells

**Figure 3:**
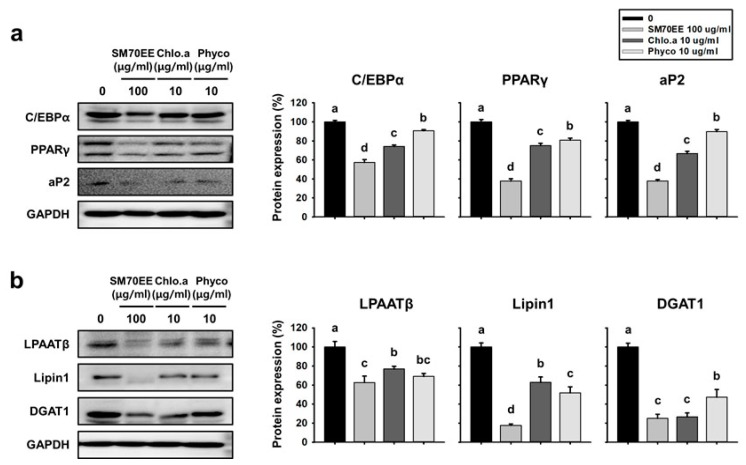
SM70EE, chlorophyll a, and C-phycocyanin reduce the expression of adipogenic and lipogenic proteins in C3H10T1/2 cells. (**a**) Western blots of adipogenic protein (C/EBPα, PPARγ, and aP2) expression after differentiation for 8 days. Quantification graphs for C/EBPα, PPARγ, and aP2 expression. (**b**) Western blots of lipogenic protein (SREBP1, LPAATβ, Lipin1, and DGAT1) expression in C3H10T1/2 cells. The specific bands were quantified and are presented as graphs. Data are expressed as mean ± SD (*n* = 3). Values with different letters are significantly different, *p* < 0.05 (a > b > c).

We next aimed to establish whether SM70EE reduces the expression of markers of adipogenesis and lipogenesis in C3H10T1/2 cells, as well as in 3T3-L1 cells. As shown in Figure 3a, the addition of SM70EE, chlorophyll a, or C-phycocyanin resulted in lower protein expression of the adipogenic factors C/EBPα, PPARγ, and aP2 than MDI treatment alone in C3H10T1/2 cells. As shown in Figure 3b, SM70EE addition in particular was associated with lower expression of the lipogenic proteins LPAATβ, lipin1, and DGAT1, but addition of chlorophyll a and C-phycocyanin also tended to have the same effect. Thus, these substances had similar effects in C3H10T1/2 cells to those observed in 3T3-L1 cells.

### 3.4. SM70EE Reduces Adiposity in HFD-Induced Obese Mice

**Figure 4:**
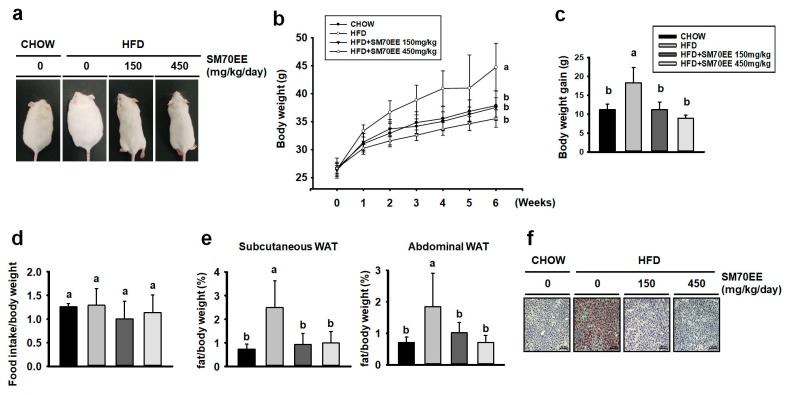
SM70EE ameliorates obesity in HFD-fed mice. (**a**) Representative image of the whole body. The effects of SM70EE on body mass (**b**), body mass gain (**C**), and food intake per unit body mass (**d**) were monitored for 6 weeks during treatment with SM70EE. (**e**) The mass of subcutaneous and abdominal fat depots per unit body mass are shown as graphs. (**f**) The effect of SM70EE on hepatic lipogenesis was assessed using oil red O staining. Mice were or were not administered with SM70EE (150 or 450 mg/kg/day) in their HFD for 6 weeks. Data are expressed as mean ± SD (*n* = 6). Values with different letters are significantly different, *p* < 0.05 (a > b).

To establish whether the inhibitory effects of SM70EE on lipogenesis and adipogenesis in vitro also occur in vivo, we analyzed their effects on the WAT of HFD-induced obese mice. Representative images of the mice are shown in Figure 4a.

In control HFD-induced obese mice, body mass rose from 26 g to 45 g over the 6 weeks of the study, but the mass gain was significantly less in SM70EE-treated HFD-fed mice (Figure 4b). From 1 week, the body weight of SM70EE-fed mice began to be significantly reduced compared with HFD-fed mice. As shown in Figure 4c, the body mass gain of HFD-fed mice treated with SM70EE was similar to that of chow-fed mice over this period. There was no difference in food consumption among the groups, suggesting that the reduction in body mass gain in SM70EE-treated mice was not due to lower food intake (Figure 4d). To clarify whether the suppression of weight gain caused by SM70EE was due to the accumulation of less adipose tissue, individual adipose depot masses were measured. As shown in Figure 4e, subcutaneous and abdominal WAT depot masses were lower in SM70EE-treated HFD-fed mice than in HFD-fed control mice. By contrast, there were no differences in the masses of other organs among the groups (Table 3).

Previous studies have shown that HFD-fed mice demonstrate greater hepatic lipid accumulation [34]. Therefore, we evaluated the effect of SM70EE on lipid accumulation in the liver of mice. Hepatic lipid droplets were identified using oil red O staining of cryosections. As shown in Figure 4F, lipid droplets were more abundant in HFD-fed control mice than in chow-fed mice, but SM70EE administration prevented excessive lipid accumulation.

### 3.5. SM70EE Administration Reduces Blood Lipid Concentrations in HFD-Induced Obese Mice

We next evaluated the effects of SM70EE supplementation on the concentrations of metabolites associated with glucose and lipid homeostasis in serum samples. It is well known that greater visceral fat accumulation is associated with higher serum TG levels and hyperglycemia in HFD-fed mice [35,36]. As shown in Table 4 and Table 5, the concentrations of serum TG and blood glucose were significantly higher in HFD-fed than chow-fed mice. However, SM70EE administration was associated with dramatically lower blood glucose and TG concentrations, indicating that SM70EE can ameliorate the hyperglycemia and hyperlipidemia induced by an HFD. Moreover, obese mice demonstrate higher rates of lipogenesis and cholesterol synthesis, which increase serum cholesterol levels [37]. SM70EE treatment was associated with lower serum concentrations of total cholesterol and LDL-cholesterol compared to treated HFD-fed mice. In addition, obesity is frequently associated with low levels of serum HDL-cholesterol [38], and HDL-cholesterol levels were lower in the HFD-fed group than the chow-fed group (Table 4), but higher in mice administered with SM70EE. Thus, our data suggest that SM70EE can prevent HFD-induced obesity and hyperglycemia.

### 3.6. Treatment with SM70EE Regulates the Expression of Markers of Adipogenesis and Browning in the WAT of HFD-Fed Mice

**Figure 5:**
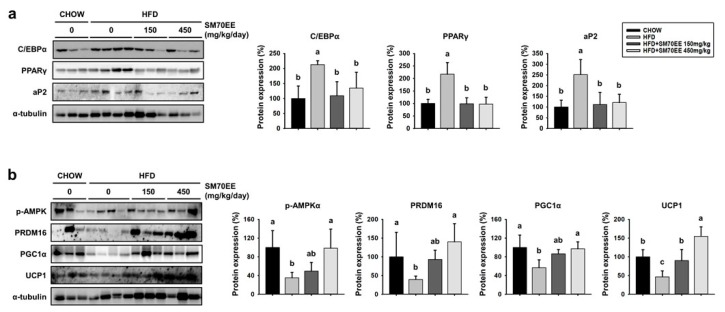
Treatment with SM70EE downregulates markers of adipogenesis and upregulates thermogenic markers in the WAT of HFD-fed mice. WAT from HFD-fed mice administered with SM70EE was analyzed for expression of adipogenic genes (**a**) and BAT-specific proteins (**b**). Specific bands were quantified and are presented as graphs. Mice were or were not administered with SM70EE (150 or 450 mg/kg/day) in their HFD for 6 weeks. Data are presented as mean ± SD (*n* = 6). Values with different letters are significantly different, *p* < 0.05 (a > b > c).

To determine whether SM70EE affected adipogenesis in WAT, we analyzed the expression of the key markers of adipogenesis C/EBPα, PPARγ, and aP2. As shown in Figure 5a, the expression of these proteins was higher in HFD-fed control mice than in SM70EE-treated mice.

In recent years, studies have shown that activation of AMPK suppresses adipocyte differentiation in 3T3-L1 cells and body weight gain in a diet-induced obese mice model by regulation of key adipogenic proteins [16,39]. Moreover, it has been revealed that phosphorylation of AMPK regulates thermogenesis, involving induction of PGC1α and UCP1 expression [40,41]. We therefore investigated whether SM70EE treatment induced trans-differentiation of white to beige adipocytes as a result of AMPK activation. As shown in Figure 5b, HFD-feeding led to lower levels of phosphorylation of AMPK and lower expression of markers of browning in WAT than were present in chow-fed mice. However, SM70EE treatment resulted in higher protein expression of the key markers of browning PRDM16, PGC1α, and UCP1, which was associated with greater phosphorylation of AMPK. Thus, the lower weight gain in the SM70EE mice appears to be caused by regulating expression of adipogenic and thermogenic proteins.

### 3.7. SM70EE Administration Upregulates Thermogenic Genes in the BAT of HFD-Fed Mice and Inducible WAT Cells

**Figure 6:**
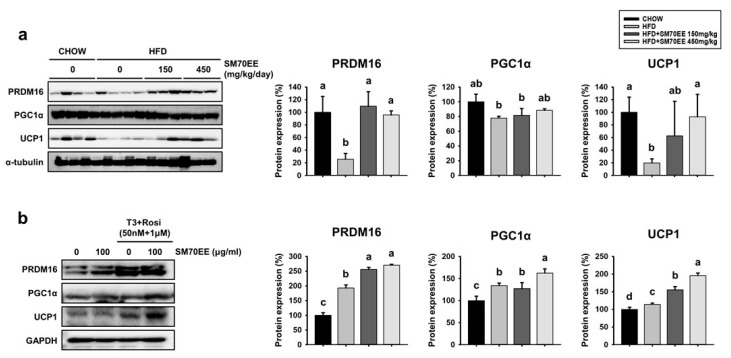
SM70EE administration upregulates thermogenic genes in BAT of HFD-fed mice and in inducible WAT cells. (**a**) BAT from mice treated with SM70EE was analyzed for the expression of BAT-specific proteins. (**b**) SVF cells were differentiated into white adipocytes, as described in the Materials and methods. Protein expression of browning factors was measured in primary WAT adipocytes. All data are presented as mean ± SD (*n* = 6). Values with different letters are significantly different, *p* < 0.05 (a > b > c > d > e).

We next wished to determine whether SM70EE treatment induces thermogenic genes not only in WAT, but also in BAT. As shown in Figure 6a, HFD-fed mice showed lower expression of key brown adipocyte-specific proteins in BAT than chow-fed mice. By contrast, SM70EE-treated mice tended to show higher protein expression of crucial BAT markers, such as PRDM16, PGC1α, and UCP1, than HFD-fed control mice. Thus, SM70EE also shows a tendency to activate thermogenesis factors in BAT.

We also wished to investigate whether SM70EE could have browning effects ex vivo. As shown in Figure 6b, gene expression analysis showed that the presence of triiodothyronine and rosiglitazone induced the expression of PRDM16, PGC1α, and UCP1 more than the differentiation induced using MDI alone. Moreover, the addition of SM70EE led to a greater expression of these markers of BAT than the absence of SM70EE groups in adipose primary cells.

## 4. Discussion

A number of studies have reported that phytochemicals can ameliorate obesity by mechanisms including the downregulation of adipogenesis and upregulation of thermogenesis [42,43]. In this study, we have demonstrated that SM70EE, chlorophyll a, and C-phycocyanin reduce lipid droplet accumulation by reducing the expression of adipogenic and lipogenic proteins in 3T3-L1 and C3H10T1/2 cells. Furthermore, we have shown that SM70EE treatment reduces body weight gain and blood lipid concentrations, most likely through downregulation of adipogenic genes. In addition, SM70EE might induce the trans-differentiation of white into brown adipocytes by increasing AMPK phosphorylation and the expression of thermogenic proteins.

In obesity, body weight increases due to accumulation of WAT. This adipose tissue accumulation is caused by both cellular hyperplasia and hypertrophy. Hyperplasia occurs through adipogenesis, in which preadipocytes differentiate into mature adipocytes, secondary to greater transcription of C/EBPα, PPARγ, and aP2 [44]. Our results indicate that SM70EE significantly reduces the expression of C/EBPα, PPARγ, and aP2 in 3T3-L1 and C3H10T1/2 cells. Expansion of WAT is also facilitated by lipogenesis, involving fatty acid synthesis and TG synthesis [45]. During adipocyte differentiation, SM70EE suppresses the synthesis of TG by reducing the expression of fatty acid and TG synthetic proteins including SREBP1, ACC, FAS, LPAATβ, lipin1, and DGAT1 in 3T3-L1 and C3H10T1/2 cells. Chlorophyll a and C-phycocyanin also attenuated fat accumulation, but SM70EE had greater anti-obesity effects in these cell types. Thus, our results suggested that consuming whole *Spirulina maxima* extract, rather than chlorophyll a or C-phycocyanin alone, may have substantial health benefits for people who are overweight or obese. Therefore, we studied the effects of SM70EE in vivo.

Consumption of an HFD increases body weight and fat mass and leads to higher circulating concentrations of TG, total cholesterol, and LDL-cholesterol, and a lower concentration of HDL-cholesterol [46,47]. In addition, hyperglycemia develops, which is a major risk factor for diabetes and other metabolic disorders [48]. Administration of SM70EE to HFD-fed mice for 6 weeks significantly suppressed body weight gain and reduced levels of blood glucose, serum TG, total cholesterol, and LDL-cholesterol, while increasing HDL-cholesterol concentrations. HFD-induced obesity and hyperglycemia has a correlation with hepatic TG accumulation [34,49]. HFD-induced hepatic lipid accumulation was diminished by SM70EE. HFD-induced adipose tissue accumulation is also associated with higher expression of C/EBPα and PPARγ [50,51], and we have shown that SM70EE-treated mice express lower levels of the adipogenic proteins C/EBPα, PPARγ, and aP2 in WAT. Thus, we made consistent findings in vitro and in vivo that imply that SM70EE treatment ameliorates defects associated with obesity.

AMPKα plays a pivotal role in metabolic regulation at the whole body level and in mitochondrial biogenesis [52]. Several studies have demonstrated that activation of AMPKα ameliorates the gain in body weight observed in HFD-induced obesity in mice [53]. For AMPKα to affect the expression of the key factors involved in mitochondrial biogenesis and energy expenditure, PGC-1α activity is also required [17]. Activation of PGC-1α induces the expression of PRDM16 and UCP1, the dominant regulator of energy expenditure [54]. UCP1 is a proton transporter that uncouples electron transport from ATP production, thereby enabling energy from the oxidized lipids to be dissipated as heat [55]. UCP1-deficient mice are heavier and have larger fat depots [56], while HFD-fed mice show lower expression of PRDM16 and PGC-1α, which are involved in thermogenesis [57,58]. In this study, we investigated whether SM70EE treatment was associated with AMPKα activation and the expression of markers of browning in WAT and BAT. Here, we have shown that SM70EE administration induces PRDM16 and UCP1 expression alongside AMPKα-PGC1α activation in HFD-fed obese mice. Thus, SM70EE treatment might lead to the differentiation of beige adipocytes by upregulating the expression of proteins of the thermogenic program, including PRDM16, PGC1α, and UCP1 in WAT and BAT. We propose that SM70EE represses obesity-related pathological factors by modulating adipogenesis and thermogenesis genes in HFD-induced obese mice. In addition, we suggest that SM70EE may be more effective than a well-known single compound chlorophyll a or C-phycocyanin with various effects.

## 5. Conclusions

SM70EE suppresses lipid accumulation by reducing the expression of key adipogenic proteins, such as C/EBPα, PPARγ, and aP2, and lipogenic proteins such as SREBP1, ACC, FAS, LPAATβ, lipin1, and DGAT1, in 3T3-L1 and C3H10T1/2 cells. The index components of SM70EE, chlorophyll a and C-phycocyanin, also inhibited adipogenesis and lipogenesis in 3T3-L1 and C3H10T1/2 cells. Moreover, SM70EE administration reduced body weight gain, fat mass, TG content, and serum total cholesterol in HFD-fed mice, implying an anti-obesity effect of SM70EE. Furthermore, SM70EE-treated HFD-fed mice showed lower expression of adipogenic proteins (C/EBPα, PPARγ, and aP2) and greater expression of thermogenic factors (PRDM16, PGC1α, and UCP1), which was likely the result of the activation of AMPKα. Therefore, our data suggest that SM70EE administration ameliorates HFD-induced obesity by inhibiting adipogenesis and activating the thermogenic expression program.

## Figures and Tables

**Table 1:** Composition of SM70EE.

Components	SM70EE (%)
**Carbohydrate**	23.51
**Crude proetin**	59.94
**Crude fat**	4.16
**Crude ash**	10.01
**Moisture**	2.38

**Table 2:** Composition of CHOW diet and HFD.

Ingredient	Group	
	CHOW (kcal%)	HFD (kcal%)
**Carbohydrate**	58	20
**Proetin**	24	20
**Fat**	18	60

**Table 3:** Effect of SM70EE administration on organ weight in HFD-fed obese mice for 6 weeks.

Variables/Groups	Organ Weight (g)			
	CHOW	HFD		
	SM70EE 0 *	SM70EE 0 *	SM70EE 150 *	SM70EE 450 *
Subcuteneous WAT	0.29 ± 0.14 ^b^	0.91 ± 0.46 ^a^	0.38 ± 0.15 ^b^	0.33 ± 0.16 ^b^
Abdominal WAT	0.32 ± 0.15 ^b^	1.66 ± 0.88 ^a^	0.34 ± 0.19 ^b^	0.38 ± 0.17 ^b^
Liver	1.55 ± 0.12 ^a^	1.47 ± 0.22 ^a^	1.42 ± 0.11 ^a^	1.38 ± 0.13 ^a^
Heart	0.21 ± 0.03 ^a^	0.19 ± 0.03 ^a^	0.19 ± 0.02 ^a^	0.18 ± 0.02 ^a^
Lung	0.20 ± 0.03 ^a^	0.22 ± 0.02 ^a^	0.22 ± 0.05 ^a^	0.22 ± 0.02 ^a^
Kidney	0.62 ± 0.06 ^a^	0.6 ± 0.04 ^a^	0.55 ± 0.07 ^a^	0.60 ± 0.05 ^a^
Spleen	0.21 ± 0.29 ^a^	0.12 ± 0.02 ^a^	0.1 ± 0.03 ^a^	0.10 ± 0.02 ^a^

* (mg/kg/day), Data are expressed as mean ± SD (*n* = 6). Values with different letters are significantly different, p < 0.05 (a > b).

**Table 4:** Effect of supplementation with SM70EE on blood lipid levels in HFD-induced obese mice.

Group	Blood Parameter (mg/dL)			
	CHOW	HFD		
	SM70EE 0 *	SM70EE 0 *	SM70EE 150 *	SM70EE 450 *
**Triglyceride**	101.6 ± 32 ^b^	164 ± 37.2 ^a^	92 ± 27.3 ^b^	99 ± 35.2 ^b^
**Total cholesterol**	129.8 ± 9.9 ^b,c^	154.5 ± 14.8 ^a^	119.3 ± 10.1 ^b,c^	135.3 ± 11.8 ^b^
**HDL cholesterol**	120.2 ± 20.2 ^a^	87.6 ± 10.4 ^c^	90.8 ± 9.9 ^bc^	104.9 ± 7.4 ^a,b^
**LDL cholesterol**	10.4 ± 3.6 ^b^	14.6 ± 4.4 ^a^	7.4 ± 1.3 ^b^	9.2 ± 1.3 ^b^

* (mg/kg/day), Data are expressed as mean ± SD (*n* = 6). Values with different letters are significantly different, *p* < 0.05 (a > b > c).

**Table 5:** Effect of SM70EE treatment on fasting blood glucose level in HFD-induced obese mice for 6 weeks.

Group	Fasting Blood Glucose (mg/dL)						
	0 week	1 week	2 week	3 week	4 week	5 week	6 week
**CHOW**	124.8 ± 12.7	136.5 ± 11.5 ^c^	122.5 ± 15.3 ^c^	117.2 ± 14.3 ^b^	116.7 ± 18.5 ^b^	107.3 ± 13.1 ^b^	102.0 ± 6.4 ^c^
**HFD**	138.2 ± 18.3	196.3 ± 17.0 ^a^	180.3 ± 12.0 ^a^	156.0 ± 39.3 ^a^	158.3 ± 26.7 ^a^	156.3 ± 27.5 ^a^	157.5 ± 16.1 ^a^
**HFD+** SM70EE 150 *	141.8 ± 21.2	173.0 ± 18.7 ^a,b^	151.7 ± 26.6 ^b^	138.7 ± 21.8 ^a,b^	144.0 ± 23.7 ^a^	123.7 ± 17.9 ^b^	121.3 ± 14.1 ^b^
**HFD+** SM70EE 450 *	129.7 ± 16.5	155.5 ± 29.6 ^bc^	150.5 ± 15.7 ^b^	130.2 ± 17.1 ^b^	136.8 ± 15.5 ^a,b^	110.3 ± 18.2 ^b^	114.2 ± 8.6 ^b,c^

* (mg/kg/day), Data are expressed as mean ± SD (*n* = 6). Values with different letters are significantly different, *p* < 0.05 (a > b > c).
